# Estimating cell type-specific differential expression using deconvolution

**DOI:** 10.1093/bib/bbab433

**Published:** 2021-10-14

**Authors:** Maria K Jaakkola, Laura L Elo

**Affiliations:** Department of Mathematics and Statistics, University of Turku, Yliopistonmäki, 20014, Turku, Finland; Turku Bioscience Centre, University of Turku and Åbo Akademi University, Tykistökatu 6, FI-20520, Turku, Finland; Institute of Biomedicine, University of Turku, Kiinamyllynkatu 10, FI-20520, Turku, Finland

## 1 Introduction

When a condition causes gene expression changes in a mixed tissue sample containing different types of cells, the changes can originate from either altered cell type composition or altered expression in some of the cell types. For example, in the context of type 1 diabetes it is an open debate if the pancreatic beta cells are dead (altered composition) or if they have, at least initially, just stopped insulin production (altered expression) [1–3]. Computational deconvolution is a free alternative to single cell and fluorescence-activated cell sorting (FACS) analyses to obtain cell type-specific information from readily analyzed bulk samples. Besides financial motivation, it also has the advantage of being applicable to old datasets, which are possibly very difficult to re-analyze in an experimental manner.

There are many publicly available tools for computational deconvolution [4–6], and they have different input requirements and goals. In deconvolution, the bulk expression of a gene is typically considered as a linear combination of its expression levels from different cell types present in the sample, i.e. $E = S \cdot C$, where $E$ is the observed bulk expression matrix with genes as rows and samples as columns, $S$ is a cell type-specific expression matrix indicating how strongly each pure cell type (columns) expresses each gene (rows), and $C$ is a cell type proportion matrix with cell types as rows and samples as columns. ‘Composition deconvolution’ (e.g. [7–12]) aims to estimate cell type composition (either proportions $C$ or abundances) in different bulk samples, whereas ‘expression deconvolution’ (e.g. [13–18]) estimates cell type-specific gene expression profiles $S$ (csGEPs). ‘Complete methods’ (e.g. [19–22]) do both tasks simultaneously.

As mentioned above, the cell type-specific expression profiles might be altered by some factors (e.g. age, gender, disease) and, in such case, the assumption of the matrix $S$ containing csGEPs as columns is overly simplistic. An important subtask related to expression deconvolution is to explore cell type-specific differentially expressed genes (csDEGs) between conditions. This can be done at three levels: associating differentially expressed genes (DEGs) detected from the bulk data with different cell types, identifying directly csDEGs, or defining csGEPs for each sample separately, i.e. personalized $S$. Personalizing cell type-specific expression is a very ambitious goal and there are only few tools available, with restrictions in their applicability. For example, ISOpure [23] is related to purifying tumor samples from the effect of immune cells, whereas CIBERSORTx [24] provides the personalization option only for datasets of limited size. Associating bulk findings with cell types is a more relaxed goal and, for instance, CellCODE [25] offers this option among other related functions. The downside of investigating bulk findings is that if cell type composition is also altered between sample groups, bulk findings are likely to contain plenty of genes that are strongly expressed by the cell type(s) with altered proportion rather than genes with altered expression in some cell types. Another issue is that genes altered in dominating cell types are likely to mask the DEGs of rare cell types at the bulk level, as discussed in [25]. Notably, csGEPs refer to columns of matrix $S$ and the term is frequently used instead of $S$ in this article. The issue with the term $S$ is that it is technically no longer a matrix, but a three-dimensional tensor or a list of matrices if it is defined for samples or sample groups separately.

Identifying csDEGs is a more relaxed goal than fully personalized $S$, but it provides more detailed insights than associating bulk DEGs with cell types. Few methods have been developed for the task [17, 25–27], but they have not been systematically investigated in the literature. Although composition deconvolution and expression deconvolution methods have been empirically compared [18, 28–30], there are no guidelines for selecting and using methods to identify csDEGs. Here, we address this issue by comparing nine different approaches, namely TOAST, csSAM, LRCDE, CARseq, Rodeo, qprog, CellDMC, TCA and DESeq2, from different practical perspectives, investigate which factors affect the accuracy and how much and offer insight when the end user can expect good results and when not.

## 2 Results

Here we evaluate nine methods for identifying csDEGs: tools for the analysis of heterogeneous tissues (TOAST) [26], cell type-specific significance analysis of microarrays (csSAM) [17], linear regression cell type-specific differential expression (LRCDE) [27], cell type aware analysis for RNA-seq data (CARseq) [31], robust deconvolution (Rodeo) [18], quadratic programming (qprog) [32], CellDMC [33], tensor composition analysis (TCA) [34] and DESeq2 [35]. Among these, TOAST, csSAM, LRCDE and CARseq are originally developed for detecting csDEGs from RNAseq data, whereas Rodeo and qprog are originally developed for expression deconvolution but we test here their utility to identify csDEGs. Methods CellDMC and TCA are originally designed for methylation data and DESeq2 represents a model not developed for deconvolution purposes of any kind. It can still be applied by defining cell type proportions and interaction terms of them and disease status as covariates. Further details about the methods and how any expression deconvolution method can be used for identifying csDEGs are available in section ‘Tested methods’. Besides accuracy, we tested the sensitivity of the methods to different factors (e.g. individual heterogeneity of csGEPs and outlier samples in the data), and how the end user can evaluate whether the obtained results are accurate.

We utilized three semi-simulated datasets addressed as GSE60424, EMTAB9221 and GSE124742 in these tests. The datasets were constructed by first generating csGEPs with realistic individual variation and DEGs between two sample groups, 100 samples each. For the evaluation purposes, these csGEPs were then combined into bulk samples by calculating their sum weighted by the cell type proportions. As gold standard csDEGs, we considered DEGs identified using the csGEPs (false discovery rate (FDR) $\leq $ 0.05). Datasets GSE60424 and EMTAB9221 are based on data from blood samples with different numbers of cell types present in them, whereas dataset GSE124742 involves measurements from pancreatic tissue samples. Further details regarding the datasets are available in section ‘Test data’.

### 2.1 Accuracy of estimated csDEGs and effect of cell type abundance

To evaluate the accuracy of the estimated csDEGs, we considered the overlap between the estimated csDEGs and the gold standard csDEGs. Among the tested methods, LRCDE detected over 5000 csDEGs with FDR $\leq $ 0.05 from all tested semi-simulated datasets and all cell types. The contrast to the other tested methods was considerable; typically the methods identified fewer csDEGs than present in the corresponding gold standard. The opposite extreme was csSAM, which did not produce any detections with FDR $\leq $ 0.05 from any cell type from any of the datasets. The exact numbers of significant findings from the different methods and cell types are available in Table S1.

Because of the large variation in the number of significant detections, we compared the top most significant findings to the gold standard. The size of the evaluated top list was defined as the number of detections in the gold standard. Table 1 lists the overlaps between the known and estimated csDEGs in the different cell types and datasets. Notably, when the FDR cutoff 0.05 was used instead of the fixed top list size, the methods typically had high precision and low recall (Section 1 in Supplementary text), suggesting that the estimated csDEGs were correct, but many true csDEGs remained undetected.

**Table 1:** The proportion of overlapping genes between the known and estimated cell type specific differentially expressed genes. The top list size, i.e. the number of gold standard detections, is indicated in the parenthesis after the cell type name. DESeq2 is designed for raw counts so it is not run for dataset GSE60424 based on normalized data and TCA threw errors we could not resolve for dataset GSE124742 so those results are missing too

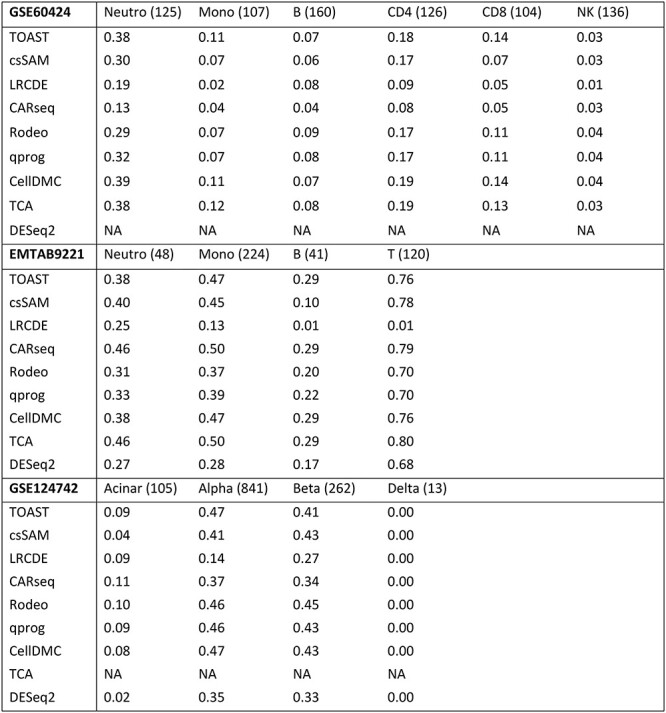

For dataset GSE60424, methods TOAST, CellDMC and TCA had the best performance. For dataset EMTAB9221, CARseq and TCA were the most accurate ones. For dataset GSE124742, TOAST, Rodeo, qprog, and CellDMC showed the best performance. Overall, LRCDE and DESeq2 provided less accurate estimates for csDEGs than the other methods, but otherwise differences between the methods’ performances were moderate as shown in Table 1.

Relative performance between the methods is not the only conclusion that can be drawn from the accuracies reported in Table 1. When the accuracies are compared against the cell type proportion information (Section 2 in Supplementary text), it can be seen that the cell type proportions affected the accuracy of the results, i.e. csDEGs from rare cell types were harder to detect than those from abundant ones. As shown in the Supplementary text, NK cells, B cells, and delta and acinar cells had the lowest average proportion in datasets GSE60424, EMTAB9221 and GSE124742, respectively. These cell types had also the lowest accuracies in the corresponding datasets (Table 1). In the case of acinar and delta cells in the GSE124742 data (both with average proportion of only 3%), delta cells were likely harder to analyze than acinar cells because they are endocrinal cells like alpha and beta cells, whereas acinar cells are from exocrinal pancreas, i.e. they likely differ more from alpha and beta cells. In Section 2 of Supplementary text, the effect of cell type abundance is investigated in a more systematic manner as we altered the average proportion of T cells in dataset EMTAB9221 and evaluated how the accuracy to detect csDEGs changed along the cell type proportion. Those results support the importance of cell type proportion and none of the methods had accuracy above 0.5 when the average proportion was decreased to 0.2. Cell type proportion has also been shown to affect the accuracy of estimated csGEPs in expression deconvolution [18].

### 2.2 Running time

Although most of the methods were fast to run (maximum few minutes), CARseq and especially expression deconvolution methods Rodeo and qprog were more time consuming. For reference, the running times with dataset EMTAB9221 for different methods were

TOAST: 3.18 s,
csSAM: 3.43 min,
LRCDE: 4.51 s,
CARseq: 1.37 h,
Rodeo: 1.74 min,
qprog: 13.21 s,
CellDMC: 28.83 s,
TCA: 2.12 min and
DESeq2: 2.27 min.

Notably, as described in section ‘Tested methods’, the running times of expression deconvolution methods Rodeo and qprog need to be multiplied by the number of random samplings for *P*-value estimation (1000 in this study) to get the full time required for detecting csDEGs. Despite making the approaches slow, the *P*-value estimation step can not be neglected as demonstrated in Section 3 of Supplementary text.

### 2.3 Individual variation affects the accuracy

Next, we investigated how the individual variation in csGEPs over samples affects the accuracy of detecting csDEGs. We set the coefficient of variation, i.e. ratio of standard deviation and mean, to a fixed level by controlling the standard deviation of the gene when constructing the csGEPs for the bulk data. Coefficients of variation of 0.1, 0.5, 1, 1.5, 2 and 2.5 were tested in dataset EMTAB9221, and they were the same for all genes. To investigate the effect of individual variation in only one cell type, we also generated data where only the coefficients of variation within monocytes were controlled while other cell types had their original, measured standard deviations varying from one gene to another. As this test was computationally intensive, only methods TOAST, csSAM, CellDMC and TCA were considered.

**Figure 1:**
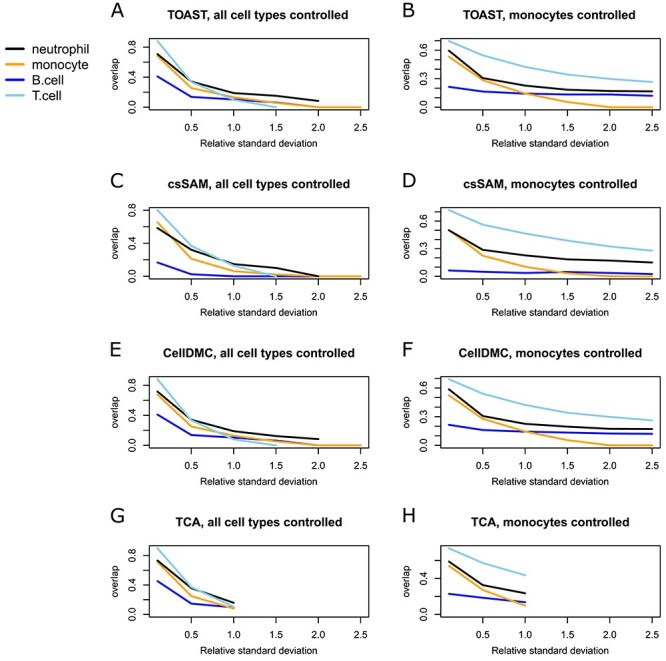
Proportion of overlapping genes between the known and estimated top csDEGs (y-axis) when the coefficients of variation (x-axis) over individuals was controlled for each cell type. The experiment was done by controlling the standard deviations of all cell types (**A**, **C**, **E** and **G**), or only of monocytes (**B**, **D**, **F** and **H**), whereas the other cell types had their original gene-specific deviation. The test was done with four methods, TOAST (**A** and **B**), csSAM (**C** and **D**), CellDMC (**E** and **F**) and TCA (**G** and **H**).

Our results show (Figure 1) that individual heterogeneity had a tremendous impact on the accuracy with all the tested methods. The impact was milder yet still considerable when only one cell type was altered (Figure 1B, D, F and H), which indicates that one very heterogeneous cell type can also weaken the results from other cell types. Similar conclusions can be drawn from dataset GSE124742 (Section 4 of Supplementary text). The measured coefficients of variation over samples had medians (over genes) of 0.93 (neutrophils), 0.48 (monocytes), 0.77 (B cells) and 0.40 (T cells) in dataset EMTAB9221 (see Section 4 of Supplementary text for coefficients of variation from all cell types and datasets). The lower coefficients of variation in T cells compared to B cells probably explain the difference in the accuracy of csDEGs detected from them (Table 1) for all the tested methods.

### 2.4 How to estimate whether the results are reliable

Our observations this far highlight the cell type proportions and internal variation over samples as important factors affecting the accuracy of the results. In addition, we tested multiple other potentially important factors, but according to our observations, their impact was minor compared to cell type proportion and individual variation. These include imbalanced sample groups (Section 5 in Supplementary text), definition of the present cell types (Section 6 in Supplementary text), and noise from very rare cell types (Section 7 in Supplementary text). Although the input matrix $C$ reveals the rare and therefore difficult to analyze cell types, individual variation in csGEPs is less straightforward to investigate. Here we provide guidelines on how to approach that issue.

**Figure 2:**
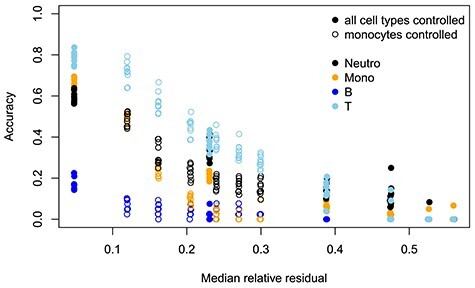
Accuracy of the csDEGs (y-axis) as a function of median relative residual (x-axis) for all cell types in dataset EMTAB9221. The multiple data points with very similar median relative residuals were from the ten randomly generated datasets with a fixed coefficient of variation.

Although the end user does not have direct knowledge about the sample heterogeneity of underlying $S$, some information can be gained by investigating residuals $|E - S\cdot C|$. In this analysis, we compared the median relative residual of a dataset (see section ’Test design’ for technical definition) to the accuracy of the identified csDEGs. Although realistically these residuals do not consist only of individual variation in csGEPs, but noise from unanalyzed cell types also contribute into them, the relative residuals still offered some guidance to estimate the accuracy of the results as shown in Figure 2. Although it is hard to define a strict median relative residual cutoff for accurate results, none of the cell types had good accuracy when the relative residuals exceeded 0.25 and quite dramatic reduction in the accuracy happened already around cutoff 0.15. Whether the change in residuals was from all cell types or from monocytes only did not have a great impact on the association between the accuracy and median relative residual for most cell types. Unsurprisingly, monocytes were an exception for that.

### 2.5 Effect of outlier samples

As the bulk data to be analyzed can contain samples, whose csGEPs drastically differ from those of the other samples, we tested how sensitive the different methods are to such outlier samples. For this purpose, one outlier case sample and five outlier control samples were added into each dataset and the accuracy of the detected csDEGs was evaluated.

**Figure 3:**
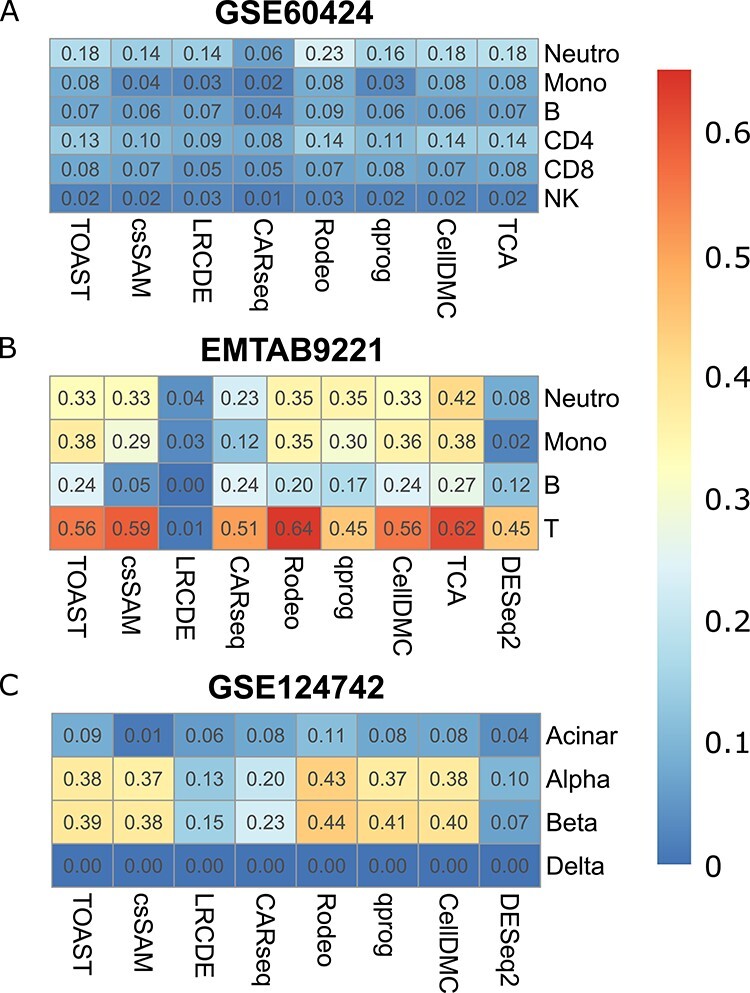
Accuracies when the datasets contain outlier samples: (**A**) GSE60424, (**B**) EMTAB9221 and (**C**) GSE124742.

Rodeo is designed to be robust against few outlier samples and indeed with such samples it had the best performance in datasets GSE60424 and GSE124742. In dataset EMTAB9221, TCA and Rodeo were the top performers. The relative performance of the other tested methods mostly did not change despite the accuracies overall decreasing, except for CARseq, which clearly suffered from the outliers in dataset EMTAB9221 and csSAM, which tolerated them fairly well in dataset GSE124742 (Figure 3).

**Figure 4:**
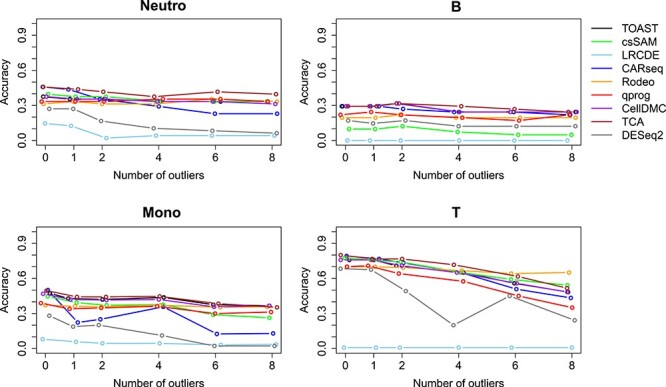
Accuracies (y-axis) of different methods when different numbers of outlier samples (x-axis) are added into dataset EMTAB9221. Jittering in x-axis is added to increase the readability of the figure.

Additionally, we tested how well the methods tolerate different numbers of outlier samples. To evaluate this, we added 1—8 outlier samples to EMTAB9221. As shown in Figure 4, Rodeo had also in this test the most robust performance, but with small number of outliers, more accurate methods still outperformed it. Notably, for most of the tested methods, accuracy of the detected csDEGs was the most vulnerable to outliers in T cells, which is the cell type with the highest accuracy (Figure 4). The other cell types’ accuracies were more robust against outliers (Figure 4).

### 2.6 Pathway analysis

As pathway-level findings can be more robust than gene-level results, we tested the reproducibility of the pathway-level results using the estimated csDEGs. TOAST and CellDMC were excluded from these tests as they do not provide fold changes (or csGEPs for both sample groups separately) required for SPIA pathway analysis, whose benefits have been shown in earlier comparisons [36, 37]. Here, we focused on cell types and datasets with >10 significant pathway findings from the known csDEGs. This left us with dataset EMTAB9221 (excluding B cells) and alpha cells from GSE124742.

None of the estimated csDEG lists generated many false positive findings, i.e. when there were only few, if any, gold standard findings, the number of findings from different methods were also low. The precision and recall values for cell types and datasets with more than 10 gold standard findings are listed in Table 2. Even in these cases, the precision values tended to be higher than the recall values, indicating that false negative findings were more of an issue than false positive findings. DESeq2 was the only method with recall higher or at least comparable with precision in most cases. Overall, similarly to the gene-level results, csSAM, CARseq, Rodeo, qrpog and TCA outperformed LRCDE and DESeq2, though for T cells in dataset EMTAB9221 DESeq2 had by far the highest recall value. The significant pathway findings are presented and discussed in Section 8 in Supplementary text.

Based on these observations, it seems that if many pathway findings can be detected from the estimated csDEGs, both pathways and the csDEGs are likely accurate. However, the opposite is not necessarily true: few or no pathway findings can indicate either inaccurate csDEGs (e.g. csSAM estimates for NK cells (GSE60424)), accurate csDEGs but no pathway findings as there is not much to find (e.g. csSAM findings for beta cells (GSE124742)), or accurate csDEGs but no pathway findings even if there would be significant pathways (e.g. csSAM findings for neutrophils (EMTAB9221)).

## 3 Discussion and conclusions

Not a single method can be claimed as the best one, but TOAST, CARseq, CellDMC and TCA had the highest accuracies. In case of over four outlier samples with altered csGEPs, Rodeo has more reliable performance. In the original publication introducing CARseq, it was validated with raw counts, which might explain why its performance compared to the other tested method was better in datasets EMTAB9221 and GSE124742, which are based on raw counts, than in GSE60424, which is based on normalized csGEPs. TCA is an accurate and fairly robust method, but it threw errors in some occasions. The observation that it performed well in sample deviation tests with small coefficients of variation but threw errors with larger variation suggests that the method does not tolerate noisy data with large heterogeneity between the samples. All methods excluding LRCDE tend to detect too few rather than too many csDEGs if FDR cutoff 0.05 is used, but the detections are mostly correct (i.e. high precision). Our results show that accurate csDEGs can be achieved if (i) the individual variation is moderate, which can be evaluated using residuals, and (ii) cell type proportion is not low. Other tested factors such as imbalanced sample groups, definition of cell types present in the samples, and minor noise from rare cell types had weaker influence on the accuracy. Pathway analysis from the estimated csDEGs can serve two purposes. Firstly, if there are fairly many (e.g. more than five) significant pathway detections, the findings are likely accurate and conclusions about underlying biological processes can be drawn as in any pathway analysis. Secondly, sufficiently many pathway findings indicate that the estimated csDEGs used as an input for the pathway analysis are accurate. However, in case there are no pathway findings, no conclusions can be drawn from that.

Notably, when estimating the accuracy of the results from the information the end user can access, we used residuals relative to bulk expression to make our conclusions robust against the overall magnitude of the bulk expression values. Also, in order to prevent possible issues caused by different depth of the measured transcriptomes, only 10 000 genes with the highest median expression in the bulk data were used to calculate the median residual. With these precautions, our cutoffs for the median residual should generalize well for other studies. However, for instance, samples from tissues with very few expressed genes might not follow the rough pattern described here.

Estimating the level of individual variation within cell types is not the only application for the residuals. It is not obvious whether the bulk data to be analyzed contains outliers, because a sample with a heavily altered cell type composition likely has a bulk expression profile clearly distinct from the rest of the samples. However, it is still not an outlier in a sense that it causes difficulties for the tested methods, unless the csGEPs are also altered. The residuals contain the variation originating from csGEPs instead of $C$, so investigating them can reveal the possible outlier samples: if the residuals of one or few samples are of greater magnitude than those of the rest of the samples, they can be identified as outliers and excluded from the bulk data and the input matrix $C$. The definition of an outlier is a distinct topic of its own. Finally, the selected method to estimate csDEGs needs to be run again without those outliers in the data.

We are unsure why results from dataset GSE60424 were less accurate than those from the other datasets despite having the lowest coefficients of variation (Section 4 in Supplementary text) over the samples for the different cell types. The dominating cell type (neutrophils) has higher internal variation than the other cell types present in the samples, but the same is true for alpha cells in dataset GSE124742, so that is not likely the key explanation. Also, the dataset has more cell types than the other test datasets (six versus four), but reducing them by combining T cells or considering NK cells as noise did not increase the accuracy. Another possible reason is that the randomly generated csDEGs in GSE60424 are weaker than those based on real observed differences within cell types in the other two datasets (section ‘Test data’). However, the number of detected gold standard csDEGs was typically similar to the other datasets, which weakens this explanation. Finally, one explanation could be the very dominating role of neutrophils, which leaves the proportions of the other cell types quite low (Section 2 in Supplementary text).

The main limitation of this study is that all our test data are semi-simulated, because cell type-specific datasets with enough sample donors were not publicly available, yet sample size has been shown to affect the accuracy of the estimated csDEGs and expression deconvolution [18, 27, 38]. Although we did our best to ensure realistic challenge in our test data, it is possible that some issues present in real measured data are missing. Such missing factors could be related to e.g. technical biases in some of the samples, which are attempted to be neutralized in normalization. In the context of deconvolution, the question of normalizing the bulk data is two-edged. One the one hand, the effect of such biases should be normalized for, but on the other hand, if a sample has particularly much cells with low overall expression level (e.g. neutrophils), its low total expression should not be normalized to a similar level to other samples. The semi-simulated test data prevents us from reliably testing the effect of normalization on the accuracy of the estimated csDEGs. It would be interesting to know if one normalization approach is generally better than the other options, or if different methods benefit from different normalizations. The magnitude of the normalization effect on the accuracy would also be of interest. In the literature, it has been suggested that transcripts per million (TPM) is a favourable normalization for composition deconvolution [39], but for expression deconvolution and especially estimating csDEGs the question is still open to our knowledge.

Besides the methods tested here, there are few tools to detect csDEGs that were excluded from this study for various reasons. For example, BSEG-sc (utilizes csSAM) [40], CellCODE [25] and PSEA [16] have input requirements that make fair comparison between them and the methods tested here difficult. Other reasons for exclusion include limitations related to the number of present cell types (e.g. Dsection [14]) and restrictions about conditions or tissues under study (e.g. DynamicDA [41]).

**Table 2:** Precision (the first value) and recall (the second value) of the pathway detections. Precision value NA indicates no findings (dividing with zero). The value in the parenthesis after the cell type name indicates the number of pathway detections from the known csDEGs. Method TCA threw errors with dataset GSE124742 and methods TOAST and CellDMC do not provide fold changes (or group specific csGEPs to calculate them) required for SPIA pathway analysis, so those results are missing

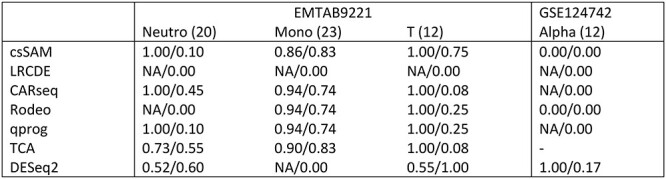

## 4 Materials and methods

### 4.1 Test data

As expression deconvolution requires more samples than available in publicly available datasets with separated cell types, we constructed semi-simulated data using three datasets, GSE60424, EMTAB9221 and GSE124742, with 200 samples. In all cases, the bulk data was constructed by first generating cell type proportion matrix $C$ and (for each sample separately) cell type-specific expression profiles in $S$, and then combining them into a bulk dataset. Thus, the bulk expression of gene $i$ in sample $j$ is constructed as

$$\begin{align*} E_{ij}=\sum\limits_{t\in T} C_{tj}\cdot S_{it}^j, \end{align*}$$

where $T$ is a set of cell types present in the sample. Cell type proportions $C$ were initially randomly generated from normal distribution (possible negative values set to 0) with the same mean and standard deviation as in the measured $C$ provided with the original public data. Then each sample was scaled to sum into 1, i.e. the random values were scaled into proportions. Construction of $S$ varied more over the datasets due to differences in the original datasets as described below, but also they imitate the realistic individual variation. Further technical details about preprocessing the single cell datasets and detecting and generating csDEGs are available in Section 9 of Supplementary text and the R codes to construct the data are available as Supplementary codes.

Dataset GSE60424 is based on RNAseq data from individuals with different autoimmune diseases available in Gene Expression Omnibus (GEO) with the same accession id [42]. In the original data, FACS separated neutrophils, monocytes, B cells, CD4 T cells, CD8 T cells and NK cells are analyzed from 20 human donors with different health conditions. Similarly to generating cell type proportions $C$, to ensure realistic individual variation in csGEPs, we generated them from normal distribution with the mean and standard deviation over the samples same as in the measured TPM normalized csGEPs.

Dataset EMTAB9221 was built from single cell RNAseq data E-MTAM-9221 [43] available in ArrayExpress. It contains cells from whole blood of eight COVID-19 patients with different severity of symptoms and healthy controls. The sample specific $S$ was built by first calculating the average expression profiles for each cell type and sample donor and multiplying them by constant 2000 to increase the general magnitude of the expression values. Then csGEPs of 200 artificial samples were generated from normal distribution with means and standard deviations following the measured csGEPs. For noise sensitivity test, we also generated a bulk data including noise from cells of unknown type by adding 10-100 (randomly selected from uniform distribution) unknown cells to the mixture.

Also dataset GSE124742 was constructed from single cell RNAseq data and the original data is available in GEO under the same accession id [44]. It contains measurements of pancreatic samples from individuals with Type 1 diabetes, Type 2 diabetes, Parkinson disease and healthy controls. From this study, we utilized data from fresh cells from healthy controls and individuals with Type 2 diabetes. We generated $S$ for each artificial individual by randomly selecting and adding 50 cells from each cell type. Gamma cells were not included due to only 41 measured cells in total. From this dataset, we generated also a bulk version including gamma cells as noise, where the number of gamma cells follows their observed proportion in the original data.

### 4.2 Test design

To evaluate the accuracy of the results, we used csDEGs detected by ROTS [45] from the known csGEPs in the generated samples with FDR cutoff 0.05 as a gold standard. Although the generated csDEGs were known, due to the randomness in the data simulation process, not all of them are necessarily detectable from the sample specific csGEPs, and therefore, only those that can be identified with ROTS are considered as a gold standard. We then calculated the overlapping proportion of these known csDEGs and an equal number of top detections from the methods as a measure of accuracy. Genes with median bulk expression 0 in both sample groups were filtered out from each dataset before the analyses. The same measure of accuracy was used in all tests. Notably, DESeq2 is designed for raw counts, so it is not run for dataset GSE60424 based on normalized csGEPs, and TCA threw errors for dataset GSE124742, so those results are missing as well.

To test the effect of individual variation in $S$, we investigated how the accuracy changed when we controlled the variation over the 200 samples. For this purpose, we constructed bulk data otherwise similarly as in the accuracy test, but the coefficients of variation of csGEPs over the samples were set to fixed levels of 0.1, 0.5, 1, 1.5, 2 or 2.5. The same level was used for all genes, which is not the case with real data. Coefficient of variation is defined as standard deviation relative to the mean, and we set it into the wanted level by controlling the standard deviation while keeping the mean expression at its measured level. Ten random samplings were done for each level of coefficient of variation and the median accuracy obtained from them is reported. For the sake of running time, this test was done only with TOAST, csSAM, CellDMC and TCA, and Wilcoxon’s test was used when extracting the gold standard. Datasets EMTAB9221 and GSE124742 were utilized for this experiment, results from EBTAB9221 are reported in this main manuscript and results from GSE124742 (reported in the Supplementary text) are used to validate our conclusions. We did this test so that the individual variation within all cell types was regulated, and so that only one cell type (monocytes in EMTAB9221 and beta cells in GSE124742) was controlled while the rest had their measured standard deviations varying over the genes.

When evaluating how the residuals can be utilized to evaluate the accuracy of the results, csSAM and data from individual variation test was used. Data with all cell types’ internal variation controlled and data with only monocytes’ internal variation controlled were both used. Method csSAM provides the residuals readily as an output, but they can be also calculated manually by subtracting re-constructed bulk $S\cdot C$ from the measured one. Absolute values of the residuals were used, as the direction of the unexplained effect was irrelevant for this test. Different normalizations and other pre-processing steps affect the general magnitude of the bulk expression values, so we used absolute residuals relative to the measured bulk expression as a measure. Median of such relative residuals was first taken over samples so that each gene has a median relative residual and then a median over top 10 000 most expressed genes was used as a summary for the dataset. Therefore, the median relative residual of a dataset is formally defined as

(1) \begin{align*} median_{G}(median_R(|E_G - S_G\cdot C|/E_G)), \end{align*}

where $G$ is the set of top 10 000 most expressed genes in $E$, $E_G$ and $S_G$ are submatrices of $E$ and $S$ containing only genes $G$, and $median_{G}$ and $median_{R}$ refer to median over gene set $G$ and median over samples, respectively.

To evaluate the effect of outlier samples, we generated one case sample and five control samples with altered csGEPs into each dataset. The cell type proportions for these samples are from the same distribution as the rest of the samples. For each cell type and data set, the csGEPs for the outliers are generated from normal distribution using randomly either lower or upper limit for an outlier as a mean. The limits for an outlier were set according to interquartile range (IQR) definition, i.e. the lower limit is $Q1 - 1.5 \cdot IQR$ and the upper limit is $Q3 + 1.5 \cdot IQR$, where $Q1$ and $Q3$ are the first and the third quartile, respectively, and $IQR = Q3 - Q1$. To evaluate the effect of the number of outlier samples on the accuracy of detected csDEGs, we added 1, 2, 4, 6 or 8 outliers into the dataset EMTAB9221.

As pathway detections can be more robust than gene-level ones, we tested their reproducibility as well. The pathway analysis was done with signaling pathway impact analysis (SPIA) [46] as its input requirements are simple and it has performed well in empirical comparison [36]. It requires fold changes of DEGs and a complete list of analyzed genes as an input. The fold changes were calculated for each cell type separately as csGEPs for case samples divided by csGEPs for control samples. In the gold standard, the sample group csGEPs were defined as median over sample specific csGEPs for both sample groups, and estimated csGEPs were readily at group-level instead of sample-level.

### 4.3 Tested methods

We tested nine methods, all implemented into R packages: TOAST (version 1.0.0), csSAM (version 1.4), LRCDE (version 1.0.1.0000), CARseq (version 0.0.0.9007), Rodeo (version 1.0), qprog (CellMix version 1.6.2), CellDMC (EpiDISH version 2.2.2), TCA (version 1.2.1) and DESeq2 (version 1.26.0). A cell type proportion matrix $C$, sample group information and a bulk expression matrix were provided as inputs for all of the methods.

Among the nine tested methods, four have been designed to detect csDEGs. TOAST [26] is a versatile deconvolution tool with various features not utilized in this study. For example, it can take into account multiple different sample characteristics (e.g. age, gender, disease status), it can perform reference free composition deconvolution, and, besides RNAseq, it has been evaluated with DNA methylation data as well. Methods csSAM [17] and LRCDE [27] are both based on linear regression. In csSAM, the significances of differences between sample group specific estimated csGEPs are estimated by randomly sampling the group labels, and in LRCDE, Welch’s two-sample *t*-test is utilized for the task. Although csSAM is an old and widely cited tool for this particular problem, LRCDE contains also a function to estimate cell type proportions using a signature matrix. CARseq [31] is a novel tool designed to detect csDEGs from count data. It uses negative binomial distribution instead of commonly utilized linear model.

Methods Rodeo and qrpog have been originally developed for expression deconvolution, but we tested here their utility to identify csDEGs. These two were selected into this study due to their accurate performance [18], but any expression deconvolution method could be utilized for detecting csDEGs as follows

1. Estimate $S$ for case and control samples separately using any expression deconvolution method.
2. Calculate (absolute) difference between $S_{case}$ and $S_{control}$. This creates a difference matrix with the same dimensions (genes as rows and cell types as columns) as in $S_{case}$ and $S_{control}$.
3. For $n$ times: randomly select the sample groups and do steps 1 and 2. Calculate how often the difference is greater than the observed one in step 2. Let $m$ be the number of times the difference exceeds the observed one.
4. Calculate *P* value estimates as $m/n$. In this study $n=1000$. This creates *P* values for all genes in all cell types. Correct *P* values into FDR values, if wanted.

Rodeo [18] is based on robust linear regression and its speciality is tolerating some outlier samples in the dataset. Qprog is based on quadratic programming and it was originally implemented as a composition deconvolution method [32], but R package CellMix [47] implements an expression deconvolution version of it, which is used in this study.

CellDCM [33] and TCA [34] are developed to estimate cell type-specific methylation profiles, but their input requirements can be met also with RNAseq data. CellDMC utilizes EpiDISH algorithm [48] and it has been demonstrated to identify also significant findings with opposite direction of regulation in different cell types. TCA is based on generalization of matrix factorization and it can consider several cofactors (e.g. age and gender) like TOAST.

DESeq2 [35] is a diverse and widely used tool to analyze RNAseq data without any particular focus on deconvolution or related topics like detecting csDEGs. Normalization is an important part of its workflow and, therefore, we have not utilized it on dataset GSE60424, which is based on normalized csGEPs. DESeq2 uses negative binomial generalized linear models and it can accommodate for user defined covariates. Here, we have used the cell type proportions and the interaction terms of them and sample group as covariates. From the output, csDEGs are then defined based on the significance of the interaction terms.

**Key Points**

- TOAST, CARseq, CellDMC and TCA have favourable performance among the tested methods, if the data does not contain many outliers.
- Methods designed for methylation data perform well also on RNAseq data.
- The most important factors affecting the accuracy of the estimated cell type-specific differentially expressed genes are (i) abundance of the cell type and (ii) individual heterogeneity between the samples.
- Investigating residuals can reveal outliers and whether the data is too heterogeneous for this type of analysis.

## Supplementary Material

SupplementaryText_bbab433

## Data Availability

This study utilizes publicly available gene expression data with either raw data and instructions to process it, or preprocessed data downloadable from GEO or ArrayExpress databases. The relevant accession ids are GSE60424 and GSE124742 for GEO data, and E-MTAB-9221 for ArrayExpress data. The R codes to further process the data are available as supplementary codes.

## Notes

**Laura Elo** is Professor of Computational Medicine and Research Director in Bioinformatics at Turku Bioscience, University of Turku. Her main research interests include computational biomedicine and bioinformatics.

**Maria Jaakkola** is a doctoral candidate working in the bioinformatics research group led by Professor Laura Elo in the University of Turku. Jaakkola's primary research interests at the time of writing are pathway analysis and deconvolution.
