# Supplementary material for: Estimating cell type-specific differential expression using deconvolution
Source: Brief Bioinform. 2021 Oct 14;23(1):bbab433. doi: 10.1093/bib/bbab433 (PMC8769698; doi:10.1093/bib/bbab433)
Supplement: SupplementaryText_bbab433 [file supplementarytext_bbab433.pdf]

Supplementary text

Estimating cell type specific differential expression using deconvolution

Maria K. Jaakkola and Laura L. Elo

1 Precision and recall

In the main manuscript we use overlaps of top detections with the top list size defined by the number of findings in the gold standard. Here we evaluate the accuracy of DE genes defined by FDR cutoff 0.05 instead of fixed number of top detections. As csSAM has no such detections from any dataset or cell type and LRCDE has thousands of them in all cases, this evaluation is limited to findings from TOAST, CARseq, Rodeo, qprog, CellDMC, TCA, and DESeq2.

Our results here (see Table S1) show that precision values are good for most cell types, especially with TOAST, but recall is much lower. This indicates that the findings with FDR cutoff 0.05 are true positives, but many significant findings are missed. The exceptions with either no significant detections or low precision are monocytes and NK cells in dataset GSE60424, and acinar and delta cells in dataset GSE124742. Among these exceptions all but monocytes of dataset GSE60424 are rare cell types, which likely makes them difficult to estimate (see section 2 in this SupplementaryText for cell type proportions).

**GSE60424**	Neutro (125)	Mono (107)	B (160)	CD4 (126)	CD8 (104)	NK (136)
TOAST	0.95/0.14/19	NA/0.00/0	0.75/0.02/4	0.80/0.06/10	1.00/0.02/2	NA/0.00/0
CARseq	0.57/0.03/7	0.00/0.00/1	0.33/0.01/6	0.33/0.01/3	0.33/0.01/3	NA/0.00/0
Rodeo	0.50/0.11/28	0.05/0.02/40	0.32/0.04/22	0.44/0.10/27	0.32/0.08/25	0.00/0.00/19
qprog	0.53/0.14/34	0.08/0.03/38	0.33/0.03/15	0.48/0.10/25	0.25/0.05/20	0.00/0.00/12
CellDMC	0.95/0.14/19	NA/0.00/0	0.80/0.03/5	0.80/0.06/10	1.00/0.05/5	NA/0.00/0
TCA	1.00/0.14/17	NA/0.00/0	0.80/0.03/5	0.88/0.06/8	1.00/0.05/5	NA/0.00/0
DESeq2	NA	NA	NA	NA	NA	NA

**EMTAB9221**	Neutro (48)	Mono (224)	B (41)	T (120)
TOAST	0.88/0.31/17	0.68/0.43/142	0.53/0.24/19	0.91/0.75/99
CARseq	0.84/0.33/19	0.95/0.34/81	0.85/0.27/13	0.95/0.73/92
Rodeo	0.65/0.27/20	0.71/0.22/69	0.29/0.17/24	0.88/0.58/80
qprog	0.58/0.29/24	0.75/0.22/65	0.25/0.20/32	0.88/0.59/81
CellDMC	0.88/0.31/17	0.63/0.44/155	0.53/0.24/19	0.91/0.75/99
TCA	0.89/0.33/18	0.74/0.46/137	0.55/0.27/20	0.95/0.75/95
DESeq2	0.23/0.31/65	0.91/0.09/23	0.57/0.10/7	0.69/0.66/115

**GSE124742**	Acinar (105)	Alpha (841)	Beta (262)	Delta (13)
TOAST	0.12/0.06/58	0.81/0.26/264	0.93/0.26/74	NA/0.00/0
CARseq	0.21/0.06/29	0.31/0.42/1123	0.48/0.29/160	0.00/0.00/23
Rodeo	0.11/0.10/103	0.77/0.20/216	0.81/0.33/108	0.00/0.00/15
qprog	0.09/0.09/99	0.79/0.19/199	0.81/0.32/102	0.00/0.00/32
CellDMC	0.11/0.07/66	0.78/0.26/283	0.94/0.28/78	NA/0.00/0
TCA	NA	NA	NA	NA
DESeq2	0.00/0.00/2	0.41/0.28/584	0.50/0.23/119	0.00/0.00/1

Table S1: Each cell contains precision, recall, and number of detections (in that order) for the cell type defined by the column and method defined by the row. The number of detections from the gold standard is indicated in the parenthesis after the cell type name.

2 Cell type proportions

Cell type proportions of the test data were generated from normal distribution with cell type means and standard deviations equal to the observed ones in the original measured data, then possible negative values were set to zero, and finally the values were scaled into proportions that sum to one. Table S2 summarizes the resulting cell type proportions for each dataset. In dataset GSE60424 neutrophils have clearly dominating proportion as compared to the other cell types, whereas in EMTAB9221 and GSE124742 also the second most common cell type (neutrophils and beta cells, respectively) has an average proportion of 25%. Notably, in dataset GSE124742 only two cell types, alpha and beta cells, heavily dominate the bulk data as acinar cells and delta cells have the average proportion of only 3%.

**GSE60424**	Neutro	Mono	B	CD4	CD8	NK
mean	0.72	0.09	0.04	0.08	0.06	0.02
sd	0.07	0.04	0.02	0.04	0.04	0.01
min	0.53	0.00	0.00	0.00	0.00	0.00
max	0.87	0.26	0.13	0.21	0.19	0.07
**EMTAB9221**	Neutro	Mono	B	T		
mean	0.25	0.11	0.07	0.58		
sd	0.15	0.04	0.04	0.14		
min	0.00	0.04	0.00	0.22		
max	0.68	0.31	0.21	0.93		
**GSE124742**	Acinar	Alpha	Beta	Delta		
mean	0.03	0.69	0.25	0.03		
sd	0.03	0.17	0.17	0.04		
min	0.00	0.24	0.00	0.00		
max	0.18	0.99	0.69	0.19		

Table S2: Mean, standard deviation, minimum, and maximum cell type proportion over samples for each cell type and dataset

In addition, we evaluated how the accuracy of estimated csDEGs from T cells in EMTAB9221 changed when their average cell type proportion is altered. T cells were selected for this test as good results were achieved from them in the main tests (Table 1 in the main manuscript), when their proportion was high (Table S2). As shown in Figure S1, cell type proportion heavily influences the accuracy of the obtained results for all the tested

methods. When the average proportion of T cells decreased into 0.2, even the best performing methods CARseq and TCA had accuracy around 0.4.

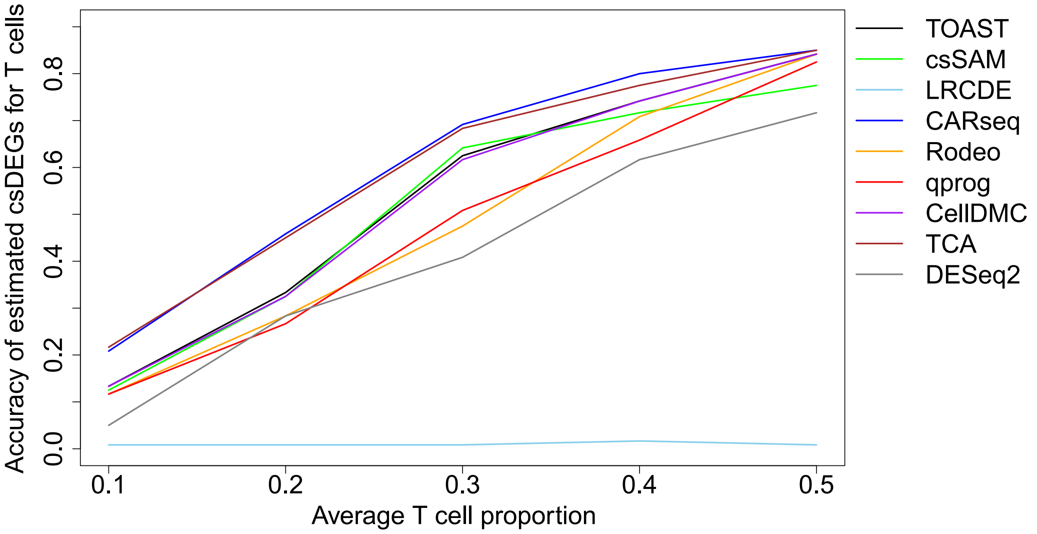

Figure S1: Accuracy of estimated csDEG (y-axis) for different methods, when the average proportion of T cells (x-axis) is altered.

3 Effect of label sampling when utilizing expression deconvolution methods

Here we evaluate how important the random sampling of group labels is when using an expression deconvolution method to estimate cell type specific differentially expressed genes (csDEGs). We tested three different criteria to rank estimated csDEGs: 1) p-value calculated with label sampling (used in the main manuscript), 2) absolute difference between S estimated for case and control samples separately, and 3) fold change between S estimated for case and control samples separately.

Our results (Figure S2) show that ranking the findings based on p-values (requires time consuming random sampling of group labels) is the best option in most of the cases. However, in dataset EMTAB9221 there were two cell types, monocytes and B cells, which got better accuracy with simple absolute difference criterion. Results with fold change based selection criterion for csDEGs were inferior to the other two approaches in all cases.

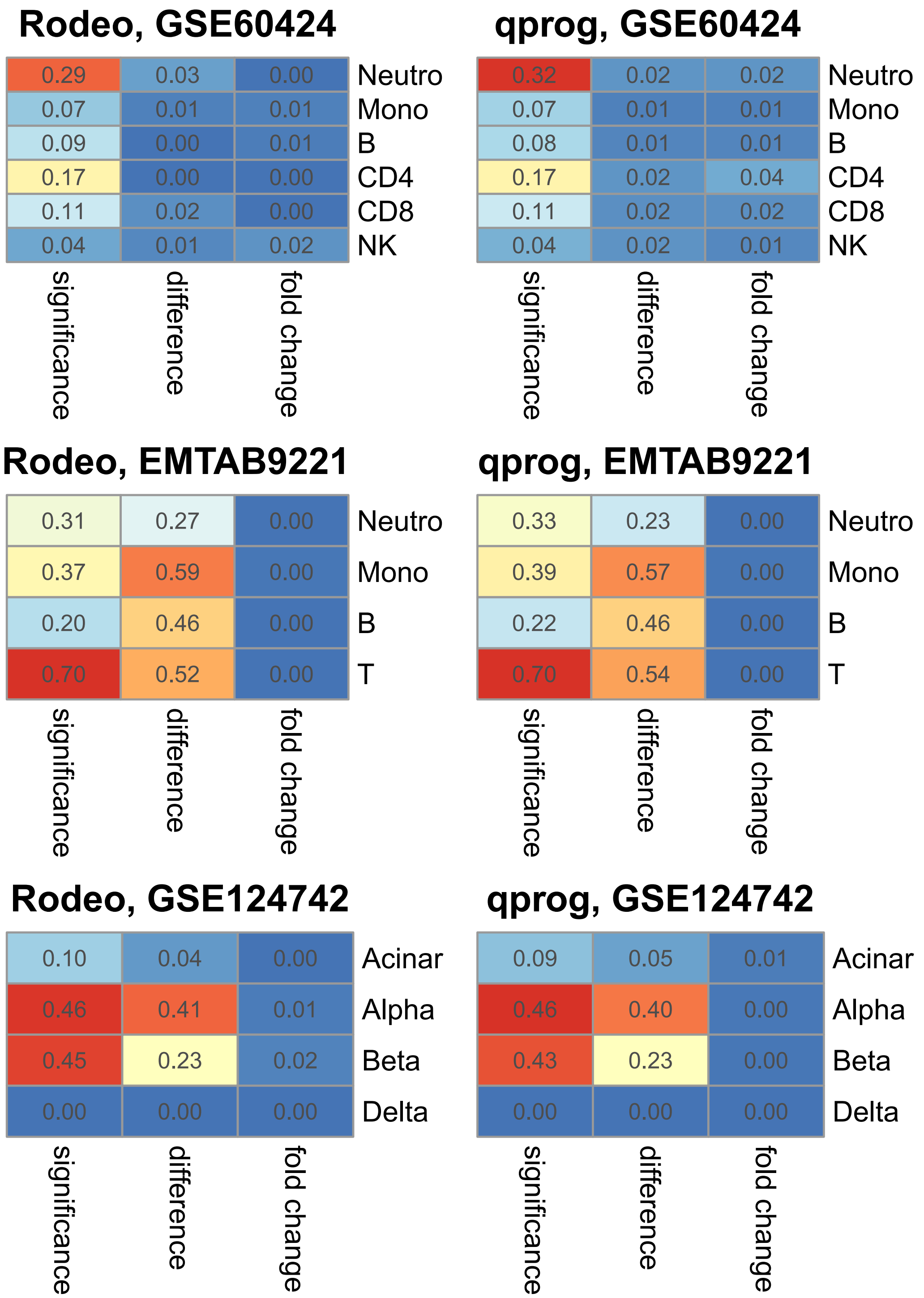

Figure S2: The overlapping proportion of estimated top detections and gold standard, with three different approaches to define cell type specific differential expression.

4 Effect of sample deviation in dataset GSE124742

In the main manuscript, we demonstrate that sample deviation has tremendous effect on the accuracy of estimated csDEGs regardless of the selected method. Here we evaluate if the effect generalizes to another dataset GSE124742. As shown in Figure S3, the conclusions remain the same: the effect is considerable and one cell type with large individual variation in S can weaken the results for all cell types.

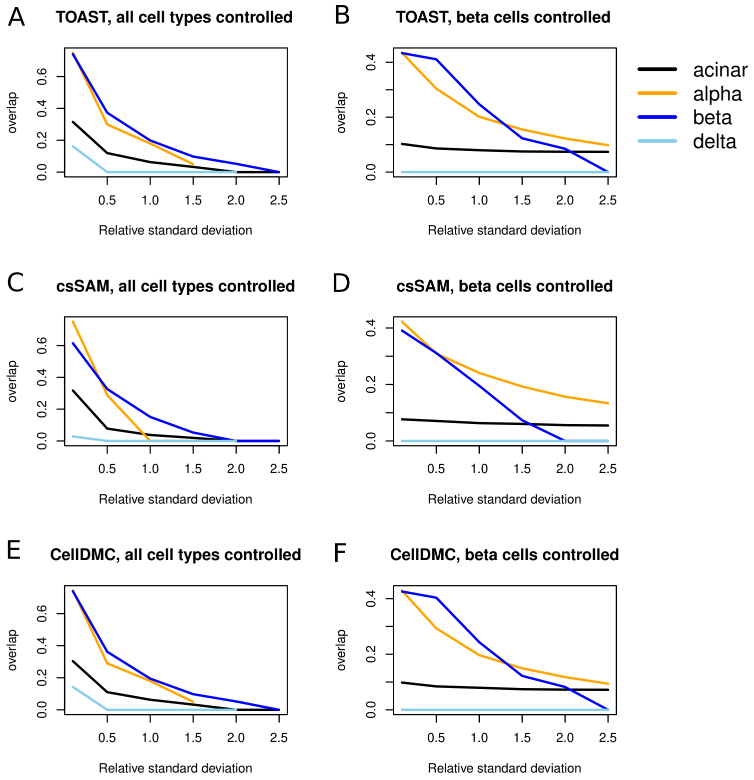

Figure S3: Overlapping proportion (y-axis) of known and estimated top csDEGs when the coefficients of variation (x-axis) over individuals was controlled within each cell type. The experiment was done so that genes' standard deviations within all cell types were controlled (A, C, and E), and only within monocytes (B, D, and F) while the other cell types had their original gene specific deviation. The test was done with three methods, TOAST (A and B), csSAM (C and D), and CellDMC (E and F).

As mentioned in the introduction of the main manuscript, both cell type composition and cell type's expression profiles contain individual variation. When generating the utilized datasets, we set the level of this sample deviation in both components to be the same as in the original measured data.

Here we present the standard deviations relative to mean expression over samples in the cell type specific expression profiles for all datasets. Notably, the standard deviations were from the original measured data, so they should represent realistic cases.

Dataset GSE60424 had overall the lowest variation in csGEPs with gene medians below 0.5 for all cell types (Figure S4A), possibly due to normalized csGEPs used to construct the bulk data. In dataset EMTAB9221 the medians were between 0.4 and 1 (Figure S4B), and in GSE124742 between 0.6 and 1 (Figure S4C).

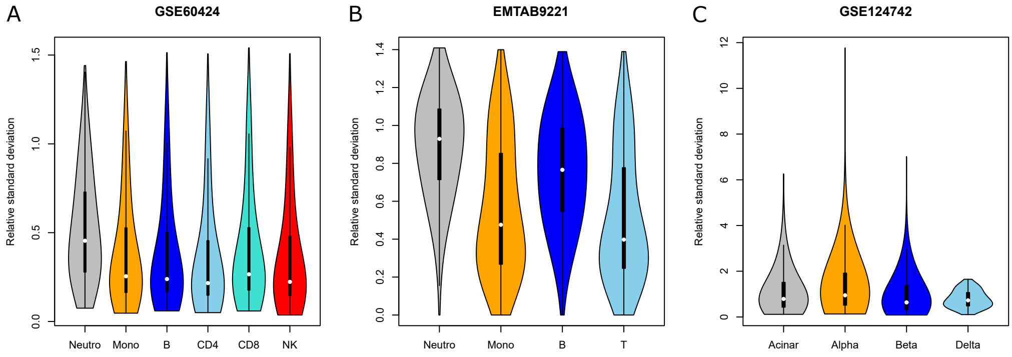

Figure S4: Standard deviations over individuals relative to genes' mean expression over individuals (y-axis) within each cell type (x-axis). Different genes form the violin plots for different cell types in datasets (A) GSE60424, (B) EMTAB9221, and (C) GSE124742.

5 Imbalanced sample groups

To test if methods' accuracies decrease if the sample groups are not balanced, as is often the case with real experiments, we generated another version of dataset EMTAB9221. The total number of samples (n= 200) is not altered, but it consists of 150 cases and 50 controls instead of 100 cases and 100 controls.

Our results show that imbalanced sample groups weaken the results in most of the cell types for all methods, but does not crush them (Table S3). Among the tested methods, accuracies of CARseq and DESeq2 results for monocytes, B cells, and T cells decreased the most due to imbalanced sample groups. The opposite extreme is increased accuracy in B cells from csSAM (from 0.10 to 0.14).

**EMTAB9221**	Neutro	Mono	B	T
TOAST	0.40 (0.38)	0.40 (0.47)	0.21 (0.29)	0.71 (0.76)
csSAM	0.33 (0.40)	0.39 (0.45)	0.14 (0.10)	0.79 (0.78)
LRCDE	0.21 (0.25)	0.12 (0.13)	0.00 (0.01)	0.01 (0.00)
CARseq	0.42 (0.46)	0.38 (0.50)	0.19 (0.29)	0.65 (0.79)
Rodeo	0.26 (0.31)	0.35 (0.37)	0.14 (0.20)	0.66 (0.70)
qprog	0.28 (0.33)	0.31 (0.39)	0.17 (0.22)	0.70 (0.70)
CellDMC	0.40 (0.38)	0.40 (0.47)	0.21 (0.29)	0.71 (0.76)
TCA	0.42 (0.46)	0.42 (0.50)	0.24 (0.29)	0.72 (0.80)
DESeq2	0.30 (0.27)	0.19 (0.28)	0.07 (0.17)	0.59 (0.68)

Table S3: Accuracies with imbalanced samples groups. The original accuracies with 100+100 samples from Table 1 of the main manuscript are available in parenthesis for comparison. Cells with accuracy difference greater than 0.05 between balanced and imbalanced sample groups are emphasised with red font.

6 Can the end user improve the results without altering the experiment?

Here we evaluate different ways the end user can improve the results. First we tested if a) combining CD4 and CD8 under T cells and b) excluding rare NK cells would improve the results from the most difficult dataset GSE60424. Notably, these changes are applied only on the input proportion matrix, not on the bulk data to be analysed. The accuracy of the estimated csDEGs was not heavily affected by these decisions related to defining present cell types nor was the effect consistent among methods or cell types (Figure S5).

Besides altering cell type proportion matrix C, we tested if the accuracy could be improved by postprocessing the estimated list of csDEGs. Namely,

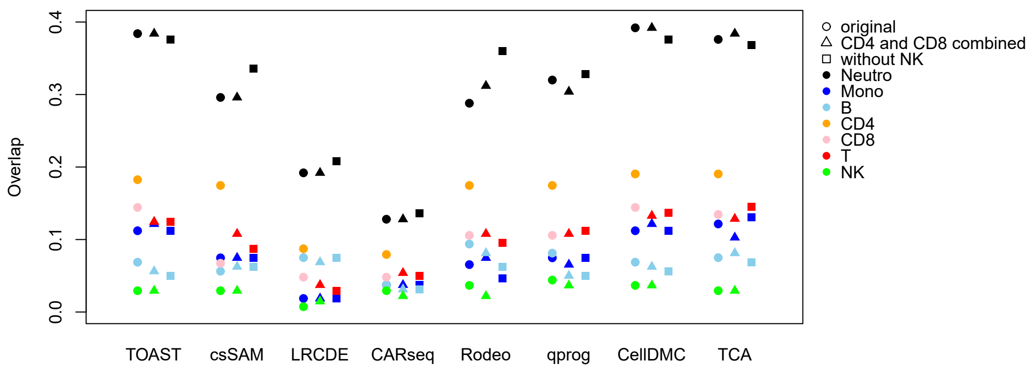

Figure S5: Overlapping proportion (y-axis) of known and estimated top cs-DEGs. For each method (x-axis) the accuracy is shown for original data with cell types Neutro, Mono, B, CD4, CD8, and NK (left-hand side), for data with CD4 and CD8 cells considered as one group of T cells (middle), and for data where rare NK cells are considered as noise rather than present cell type (right-hand side). Also in the last case the CD4 and CD8 are combined into T cells.

we excluded lowly expressed genes (according to estimated S) from the detections to see if it increases the overlapping proportion of known and estimated top detections. We tested cutoff values from 0.05 to 0.70 defining the percentage of genes with lowest expression to be filtered out. While the results for some cell types and methods benefited from the filtering, in most of the cases any filtering had either negative impact or no major impact on the accuracy of the results. Especially the accuracies of TOAST and CellDMC results decreased drastically in many cases if filtering was done (Figure S6).

These results indicate that the possibly tricky task of defining present cell types does not have critical impact on the accuracy of the results. On the other hand, the end user can not reliably improve the accuracy of the results by filtering out detections with low expression in estimated S.

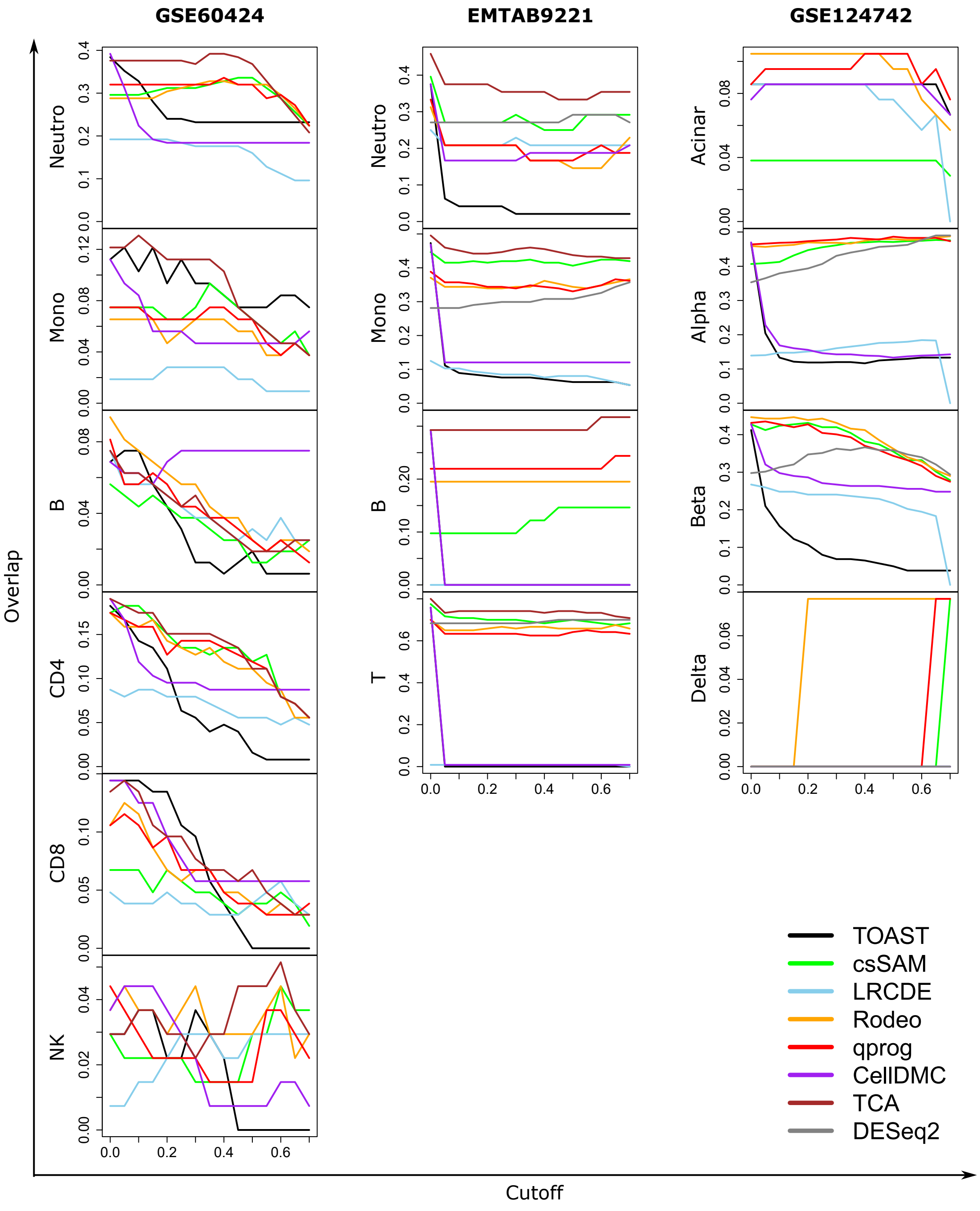

Figure S6: Overlapping proportion (y-axis) of known and estimated top cs-DEGs when lowly expressed estimated detections are filtered out with different cutoffs (x-axis). CARseq is missing due to the output not containing matrix S for defining the most lowly expressed genes in each cell type.

7 Noise from other cell types

Besides individual variation in analysed cell types, also small amounts of other cell types not present in C generate some noise into bulk data. We test the effect of such noise by analysing noisy versions of the datasets with different methods. In dataset EMTAB9221 the noise comes from small amounts of unidentified cells and in dataset GSE124742 it is from gamma cells. See further details from section 4.1 of the main manuscript describing datasets. Corresponding results from dataset GSE60424 with NK cells considered as noise are available in section 6 of this SupplementaryText where the end user's possibilities to affect the accuracy by e.g. defining the analysed cell types is investigated.

Differences in accuracy obtained with and without noise from other cell types were below 0.05 in all cases (Figure S7). This indicates that none of the tested methods are sensitive to minor presence of unanalysed cell types, which is important as tissue samples are rarely absolutely pure.

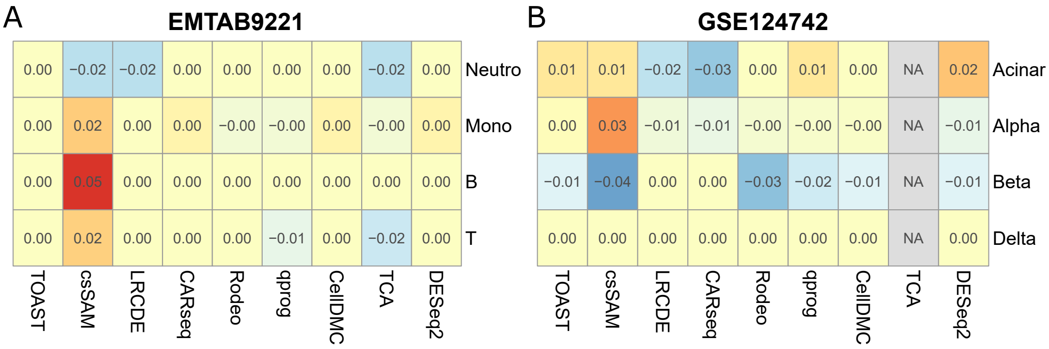

Figure S7: Difference in accuracy with and without noise from other cell types in datasets (A) EMTAB9221 and (B) GSE124742.

8 Pathway findings

Here we present the pathway findings detected using the gold standard cs-DEGs (see Section Pathway analysis from the main manuscript). Table S4 lists the detected pathways from the four cell types with more than 10 significant pathway findings.

**Neutro/EMTAB9221**	**Mono/EMTAB9221**
Cytokine-cytokine receptor interaction (S, C, q, T)	Antigen processing and presentation (S, C, R, q, T)
NF-kappa B signaling pathway (C, T, D)	Rheumatoid arthritis (S, C, R, q, T)
Amoebiasis (C, T)	Type I diabetes mellitus (S, C, R, q, T)
MAPK signaling pathway (C, T, D)	Graft-versus-host disease (S, C, R, q, T)
Chemokine signaling pathway (T)	Staphylococcus aureus infection (S, C, R, q, T)
Serotonergic synapse (D)	Allograft rejection (S, C, R, q, T)
Tuberculosis (D)	Autoimmune thyroid disease (S, C, R, q, T)
Epithelial cell signaling in Helicobacter pylori infection (C, T, D)	Asthma (S, C, R, q, T)
Retrograde endocannabinoid signaling (-)	Leishmaniasis (S, C, R, q, T)
Malaria (-)	Intestinal immune network for IgA production (S, C, R, q, T)
Toll-like receptor signaling pathway (T, D)	Viral myocarditis (S, C, R, q, T)
Antigen processing and presentation (D)	HTLV-I infection (S, C, R, q, T)
NOD-like receptor signaling pathway (C, T)	Systemic lupus erythematosus (S, C, R, q, T)
Salmonella infection (C, q, T, D)	Influenza A (S, C, R, q, T)
Osteoclast differentiation (C, D)	Herpes simplex infection (S, C, R, q, T)
Pertussis (S, C, T, D)	Toxoplasmosis (S, C, R, q, T)
Morphine addiction (-)	Tuberculosis (S, C, R, q, T)
Leishmaniasis (T, D)	Epstein-Barr virus infection (S)
Dopaminergic synapse (-)	African trypanosomiasis (-)
Chagas disease (American trypanosomiasis) (D)	NOD-like receptor signaling pathway (S, T)
	NF-kappa B signaling pathway (-)
	Malaria (T)
	Osteoclast differentiation (-)
**T/EMTAB9221**	**Alpha/GSE124742**
Herpes simplex infection (S, C, R, q, T, D)	Hepatitis C (D)
Antigen processing and presentation (S, D)	Herpes simplex infection (D)
Viral myocarditis (D)	Influenza A
Viral carcinogenesis (S, R, q, D)	Vibrio cholerae infection
Natural killer cell mediated cytotoxicity (S, D)	Salmonella infection
Allograft rejection (S, D)	Epstein-Barr virus infection
Autoimmune thyroid disease (S, D)	Viral carcinogenesis
Type I diabetes mellitus (S, D)	Protein processing in endoplasmic reticulum
Graft-versus-host disease (S, D)	Toxoplasmosis
Epstein-Barr virus infection (D)	RNA transport
T cell receptor signaling pathway (S, R, q, D)	Pathogenic Escherichia coli infection
HTLV-I infection (D)	Pancreatic secretion

Table S4: Significant pathways from neutrophils (EMTAB9221), monocytes (EMTAB9221), T cells (EMTAB9221), and alpha cells (GSE124742). Letters in the parenthesis after pathway names indicate which methods managed to identify the pathway. The mapping between the letters and the methods is S=csSAM, L=LRCDE, C=CARseq, R=Rodeo, q=qprog, T=TCA, and D=DESeq2. Results for TOAST and CellDMC are missing as explained in the main manuscript.

The detected pathways contain particularly many viral infection and autoimmune disease related findings, which is not unexpected considering that the comparison is severe COVID-19 vs healthy in EMTAB9221 and Type 2 diabetes vs healthy in GSE124742. In the recent literature, similarities between severe COVID-19 infection and autoimmune diseases (e.g. [1]) and the possibility of severe COVID-19 causing autoimmune disease alike conditions (e.g. [2, 3]) have been suggested. However, the many autoimmune disease related pathway findings from monocytes and T cells of dataset EMTAB9221

might origin also from high proportion of diabetes patients among the blood donors with severe COVID-19 in the original data used to construct dataset EMTAB9221 [4]. Also viral infections and Type 2 diabetes have been strongly associated in the literature [5,6]. Particularly Hepatitis C has been linked to Type 2 diabetes [7–9], and the corresponding pathway is indeed among the significant findings from alpha cells when T2D patients have been compared to healthy controls (dataset GSE124742). Notably, some of the findings are directly relevant for the analysed tissue or cell type. Such examples are 'T cell receptor signaling pathway' detected from T cells and 'Pancreatic secretion' detected from alpha cells.

9 Details about constructing the test data

Original single cell datasets EMTAB9221 and GSE124742 contained unknown cells besides annotated ones. We tried to identify some of the unknown cells by constructing a log2 median profile over all readily annotated cells for each cell type and investigating correlations and euclidean distances between them and unknown cells. If an unknown cell had the highest correlation and the lowest distance with the same cell type's median profile, it was annotated to that. Like this we were able to annotate 767 out of 1220 unknown cells in EMTAB9221 and 3833 out of 6726 in GSE124742. Before constructing csGEPs for GSE124742 as described in the main manuscript, we excluded cells labelled as empty wells or having total raw expression below 10000 and number of expressed genes below 100.

Original dataset GSE60424 did not include two suitable sample groups with sufficient sample sizes for analysis of differential expression, so csDEGs were randomly selected in that case. We generated 200 randomly selected differentially expressed genes into each cell type by multiplying the mean and standard deviations with a randomly selected scaling factor for half of the samples. This creates two artificial sample group of 100 samples. In the other two datasets the csDEGs were generated according to detected csDEGs in measured samples (control vs severe case in EMTAB9221 and control vs Type 2 diabetes in GSE124742), and the known means and standard deviations of the measured sample groups were utilized to generate similar csDEGs into the test data with 200 samples. The known csDEGs were identified with ROTS.

References

[1] Sahar Najafi, Elham Rajaei, Rezvan Moallemian et al. The potential similarities of COVID-19 and autoimmune disease pathogenesis and therapeutic options: new insights approach. Clinical Rheumatology 2020;:1–13.

[2] M Mateu-Salat, E Urgell, and A Chico. SARS-COV-2 as a trigger for autoimmune disease: report of two cases of Graves disease after COVID-19. Journal of endocrinological investigation 2020;43:1527–1528.

[3] J Amiral. Can COVID-19 Induce an autoimmune disease associated with long-lasting symptoms and delayed complications. Ann Clin Immunol Microbiol 2020;1014:.

[4] Aymeric Silvin, Nicolas Chapuis, Garett Dunsmore et al. Elevated calprotectin and abnormal myeloid cell subsets discriminate severe from mild covid-19. Cell 2020;182:1401–1418.

[5] Tamara Turk Wensveen, Dora Gašparini, Dario Rahelić et al. Type 2 diabetes and viral infection; cause and effect of disease. Diabetes research and clinical practice 2021;172:108637.

[6] Sajjad Karim, Zeenat Mirza, Mohammad A Kamal et al. An association of virus infection with type 2 diabetes and Alzheimers disease. CNS & Neurological Disorders-Drug Targets 2014;13:429–439.

[7] Alessandro Antonelli, Clodoveo Ferri, Poupak Fallahi et al. Hepatitis C virus infection: evidence for an association with type 2 diabetes. Diabetes care 2005;28:2548–2550.

[8] Cho Naing, Joon Wah Mak, Syed Imran Ahmed et al. Relationship between hepatitis C virus infection and type 2 diabetes mellitus: meta-analysis. World journal of gastroenterology: WJG 2012;18:1642.

[9] Francesco Negro and Mahnaz Alaei. Hepatitis C virus and type 2 diabetes. World journal of gastroenterology: WJG 2009;15:1537.
